# Non-visual effects of diurnal exposure to an artificial skylight, including nocturnal melatonin suppression

**DOI:** 10.1186/s40101-019-0203-4

**Published:** 2019-08-28

**Authors:** Akira Yasukouchi, Takafumi Maeda, Kazuyoshi Hara, Hiroyuki Furuune

**Affiliations:** 1 Department of Human Science, Faculty of Design, Kyushu University, 4-9-1, Shiobaru, Minami-ku, Fukuoka, 815-8540 Japan; 2 La Forêt Engineering Co., Ltd, Roppongi Annex 7F, 6-7-6, Roppongi, Minato-ku, Tokyo, 106-0032 Japan

**Keywords:** Artificial skylight, Fluorescent light, Office, Non-visual effect, Arousal level, Melatonin secretion

## Abstract

**Background:**

Recently, more consideration is being given to the beneficial effects of lighting on the maintenance and promotion of the health and well-being of office occupants in built environments. A new lighting technology using Rayleigh scattering has made it possible to simulate a blue sky. However, to date, no studies have examined the possible beneficial effects of such artificial skylights. The aims of this study were to examine the non-visual effects of artificial skylights and conventional fluorescent lights in a simulated office environment and to clarify the feature effects of the artificial skylights.

**Methods:**

Participants were 10 healthy male adults. Non-visual effects were evaluated based on brain arousal levels (*α*-wave ratio and contingent negative variation [CNV]), autonomic nervous activity (heart rate variability [HRV]), work performance, and subjective responses during daytime exposure to either an artificial skylight or fluorescent lights, as well as nocturnal melatonin secretion.

**Results:**

Subjective evaluations of both room lighting-related “natural” and “attractive” items and the “connected to nature” item were significantly higher with the skylight than with the fluorescent lights. Cortical arousal levels obtained from the early component of the CNV amplitude were significantly lower with the skylight than with the fluorescent lights, whereas *α*-wave ratio and work performance were similar between the two light sources. The HRV evaluation showed that sympathetic nerve tone was lower and parasympathetic nerve tone was higher, both significantly, for the skylight than for the fluorescent lights during daytime. Nocturnal melatonin secretion was significantly greater before and during light exposure at night under the daytime skylight than under the fluorescent lights.

**Conclusions:**

Our results suggest that artificial skylights have some advantages over conventional fluorescent lights in maintaining ordinary work performance during daytime with less psychological and physiological stress. The findings also suggest that the artificial skylights would enable built environments to maintain long-term comfort and productivity.

## Background

Physiological anthropology is a field that primarily focuses on humans living in modern society. This is because humans are considered to be adapted to ancient environments, given that we spent nearly all of our history as a species in such environments, dating back to our hunter-gatherer stage. Thus, there must be a discrepancy between environments to which humans have already adapted and environments to which humans are struggling to adapt when seeking comfort and convenience. For this reason, physiological anthropologists study the environmental pressures on humans in highly technological environments [1–6], in which lighting must also be examined as one of the physical factors affecting environmental adaptability [7–9].

Windows give occupants in built environments feelings of connectedness to nature and help them to feel more relaxed, which boosts their health and well-being [10–13]. However, occupants in windowless environments or those who are situated away from windows have to depend on artificial light and have no connection to nature. Natural light and views from windows also enhance attention and cognition [14], whereas windowless environments or those with relatively low artificial light illumination negatively affect arousal level and productivity due to sleepiness [15]. Furthermore, there is no time-dependent variation in natural light and views throughout the day in windowless environments, which can increase psychological stress [16] and lower job satisfaction [17].

In addition to more advanced lighting technologies such as light-emitting diodes (LEDs) and organic electroluminescence (OEL), a new technology has been developed to simulate natural sunlight and blue skies. This system is composed of a light source and nanostructured material that recreates the Rayleigh scattering phenomenon that occurs naturally in the Earth’s atmosphere whereby shorter wavelengths of light are strongly diffused while longer wavelengths are weakly diffused. This phenomenon can be recreated in a small box and helps people to perceive a realistic sun and blue sky with indefinite depth, even though the spectral distribution is not exactly the same as that of natural sunlight. The introduction of this artificial skylight in a windowless environment or space situated away from windows gives the impression of connectedness to nature, which should boost psychological and physiological well-being and health.

Canazei et al. [18] determined, using a questionnaire-based approach, that an artificial skylight similar to the one used in this study had beneficial effects compared with conventional fluorescent lighting. However, in addition to psychological effects, physiological responses caused by non-visual effects or non-image-forming effects should also be considered to improve worker health, well-being, and productivity [15, 19–23]. Ours is the first study to examine the non-visual effects of artificial skylights in a simulated office environment. The existence of non-visual effects was suggested when the retinohypothalamic tract was first identified [24] and, as a non-visual effect, light-induced nocturnal melatonin suppression was first reported by Lewy et al. [25]. Brainard et al. [26] and Thapan et al. [27] have reported the peak spectral sensitivity of light-induced melatonin suppression at night, with the peak located at around the blue part of light, which was soon further supported by the discovery of intrinsically photosensitive retinal ganglion cells (ipRGCs) [28]. There are several important neural sites from the retina to the pineal body via the hypothalamus that are related to circadian rhythms, autonomic nervous activity, endocrine secretion, cortical arousal level, and muscle tension. These are called “non-visual effects” and are associated with signal input to ipRGCs projected to the retinohypothalamic tract. The intensity, wavelength, duration, timing, and pattern of light are important factors affecting photic resetting of non-visual effects [29], particularly in terms of the circadian system and suppression of melatonin secretion. Thus, changes in physiological function caused by the light environment would affect the productivity, comfort, and well-being of office workers, not only as an acute effect, but also as a long-term effect.

The aims of this study were to examine the cortical arousal level, autonomic nervous activity, and nocturnal melatonin secretion, as well as work performance and subjective evaluations, of participants exposed to artificial skylights and conventional fluorescent lights in a simulated office and to clarify the feature effects of the artificial skylight.

## Methods

### Participants

Participants were 10 healthy male college students (mean age ± standard deviation, 23.6 ± 0.7 years). They were enrolled in the study after completing a psychological interview and screening for sleep disorders, medication use, recent night work, and recent overseas travel across time zones. This study was approved by the Ethics Committee of the Faculty of Design, Kyushu University. Written informed consent was obtained from all participants prior to enrollment. Participants were required to keep a regular 8-h sleep schedule for at least 5 days before the experiment, which was confirmed from their daily sleep diary and actigraph data (Actiwatch-L; Mini-Mitter Co., Inc., Bend, OR). Participants were also asked to refrain from using caffeine, nicotine, and alcohol for 1 day before the experiment.

### Experimental protocol

**Fig. 1:**
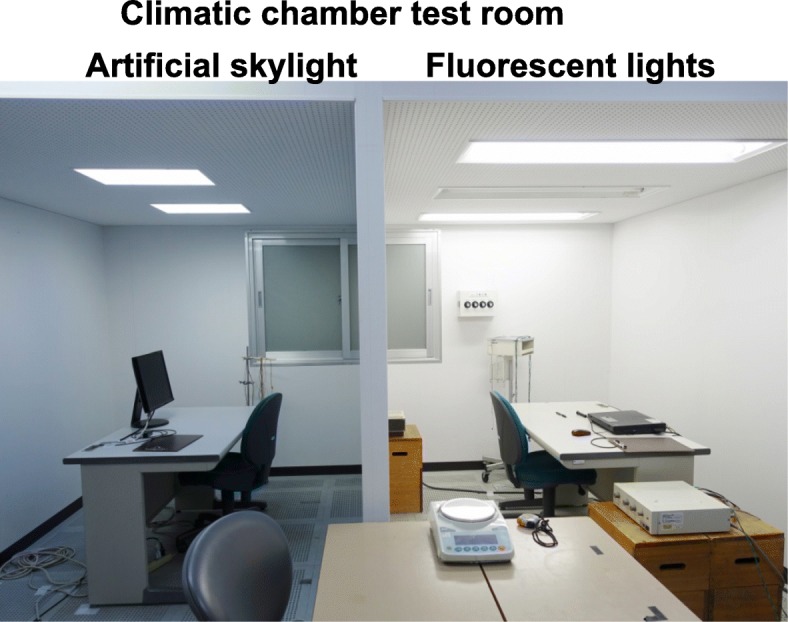
On the left is the room with artificial skylights; the room with fluorescent lights is shown on the right

A climatic chamber at the Faculty of Design, Kyushu University, was used for our study from November to December 2017. Two simulated office environments with identical areas (width, 1960 mm; length, 2700 mm; height, 1940 mm) were set up as shown in Fig. 1. The ambient temperature in each room was kept at 25 ± 0.04 °C with 50 ± 0.10% relative humidity. The artificial skylight room (CoeLux ST, CoeLux S.r.L) is shown on the left side of the figure, and the fluorescent lightroom is shown on the right side.

**Fig. 2:**
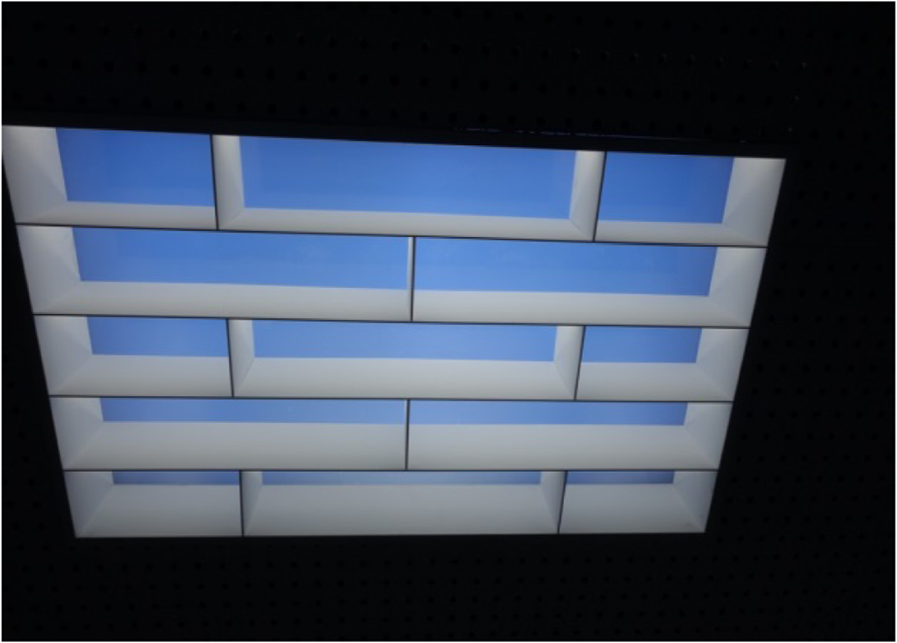
Artificial skylight seen from below

**Fig. 3:**
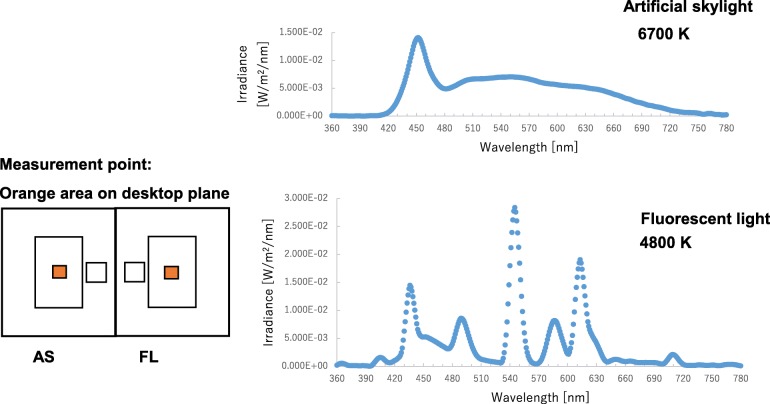
Spectral distribution of the artificial skylight (upper) and the fluorescent lights (lower). The wavelength region around 480 nm is where photoreceptors related to non-visual effects (ipRGCs) have high sensitivity. AS, artificial skylight; FL, fluorescent lights

The lighting conditions of illuminance and correlated color temperature (CCT) when horizontal illuminance was set as the same light intensity measured on the desk (440 lx) are described in Table 1. The work plane is where the most important tasks in the office occur and because the relevant guidelines in each country generally recommend that horizontal illuminance be measured on the desk. The melanopic illuminance obtained from corneal spectral irradiance measures calculated by the equation of Lucas et al. [30] determined 4.99 melanopic lx with skylights, which is about 35% greater than the 3.70 melanopic lx with fluorescent light. In addition, cyanopic, rhodopic, chloropic, and erythropic illuminances were also 22%, 22%, 8%, and 0% higher with skylights, respectively. An artificial skylight installed on the ceiling is shown in Fig. 2, and the spectral power distributions of the skylights and fluorescent lights are shown in Fig. 3.

**Table 1:** Lighting conditions of the artificial skylights and fluorescent lights

	Measured on the desk		Measured at the cornea	
	Illuminance (lx)	CCT (K)	Illuminance (lx)	CCT (K)
AS	440	6700	417	6200
FL	440	4800	330	4800

*CCT* correlated color temperature, *AS* artificial skylight, *FL* fluorescent lights

**Fig. 4:**
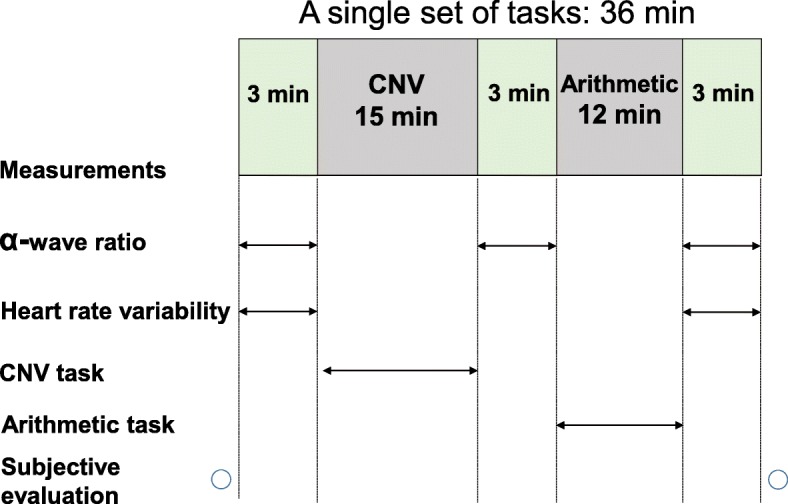
Simulated office work during daytime

**Fig. 5:**
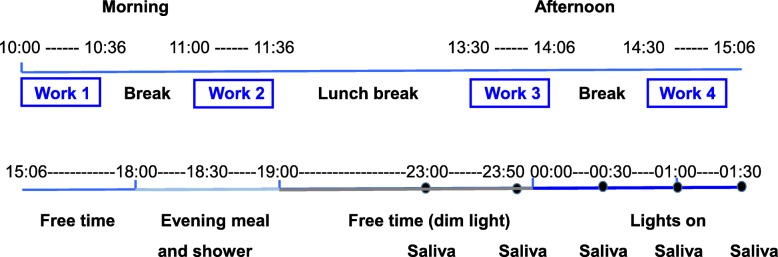
Schedule lighting conditions: 09:00–18:00: artificial skylight or fluorescent lights (see Table 1). 18:00–19:00: artificial skylight or fluorescent lights with 200 lx and CCT kept the same; 19:00–00:00: dim light condition (< 30 lx). 00:00–01:30: fluorescent lights with 500 lx and 5000 K

All measurement items and timings are shown in Fig. 4, and the schedule is shown in Fig. 5. Participants arrived at the laboratory before 09:00 and wore t-shirts and shorts to homogenize the thermal conditions.

After we set the sensors to take measurements until 09:50, a subjective evaluation of the lighting in the room was made and the first task was started. The simulated office tasks consisted of a contingent negative variation (CNV) paradigm and arithmetic task. Both tasks were performed for 36 min with a 3-min rest and interval as a single set (Fig. 4). The set of tasks was performed twice each morning and twice each afternoon (Fig. 5). The measurement timing of *α*-wave ratio, heart rate variability (HRV), and subjective evaluation in a set are shown in Fig. 4. The early component of the CNV amplitude and the selective response time were measured during the CNV paradigm, and the number of answers and ratio of correct answers were obtained during the arithmetic task. HRV was not measured before the CNV paradigm in the second set each morning and afternoon.

As shown in Fig. 5, the first and the second task sets started at 10:00 and 11:00 in the morning and at 13:30 and 14:30 in the afternoon, respectively, and were followed by free time until 18:00. The participants were permitted to read books, watch videos, and play games that did not overly stimulate them during the free-time periods after 15:00 and 19:00. The display was set to 0.5 cd/m^2^ during the videos and games so as to not affect melatonin secretion, in accordance with Higuchi et al. [31]. The lighting conditions were as shown in Table 1 during the period of office simulation from 10:00 to 18:00, set at 200 lx measured on the desk for each light source shown in Table 1 during the evening meal and shower period from 18:00 to 19:00, and emitted dim light (< 30 lx) during the free period from 19:00 to 00:00. Saliva samples were taken five times (solid circles in Fig. 5) from 23:00 to 01:30 for analysis of melatonin concentrations. During this period, participants, regardless of the light source condition during the daytime, were exposed to fluorescent light at 500 lx and 5000 K from 00:00 to 01:30 to evaluate light-induced nocturnal melatonin suppression. The experiment was then finished.

### Measurements

#### Contingent negative variation

CNV, one of the event-related potentials in the brain, was first proposed by Walter et al. [32], who suggested that the components of the CNV reflect attention and expectancy related to a given stimulus as well as arousal level as a fundamental function. In this study, electroencephalography (EEG) was measured by using Ag-AgCl disc electrodes located at Fz, Cz, and Pz according to the International 10–20 system with a linked ear reference (Polimate AP1000; TEAC Co. Ltd., Tokyo, Japan). The CNV paradigm consisted of a warning stimulus (S1) and imperative stimulus (S2) with a 2500-ms interval. The paradigm was repeated 44 times over 15 min, and the inter-trial interval was randomized between 8000 and 12000 ms. The baseline level was obtained from the averaged potential during the 500 ms before the S1 stimulus. The arithmetic mean of the early component of the CNV amplitude was obtained between 500 and 1000 ms from the S1 stimulus during the 44 trials with the exclusion of trials with noise due to eye movement or blinking detected by electrooculography. The imperative stimulus (S2) consisted of a red circle as a target signal and a green circle as a non-target signal; target signals appeared in 70% of all trials. The participant was requested to push the button as soon as possible upon recognizing the red signal of S2 on the display.

#### Work performance

The average response times of correct answers during the CNV paradigm were considered measures of work performance. The arithmetic task was a single-digit addition task in which participants were asked to add neighboring digits for 12 min, and the number of answers and the ratio of correct answers were considered to reflect work performance.

#### *α*-wave ratio

The spontaneous EEG at Pz was measured for 3 min (Fig. 4) with participants at rest sitting on a chair with their eyes open. The power values for the *α*-wave (8–13 Hz) and *β*-wave (13–30 Hz) were calculated from a fast Fourier transform (FFT) of the EEG data. The relative power value of the *α*-wave (*α*-wave ratio) was obtained by *α*rp/(*α*rp + *β*rp) × 100, where rp is the relative power value.

#### Heart rate variability

Participants were asked to gaze at a point on the wall in front of them. Their breathing was controlled at 15 breaths/min with a metronome while an electrocardiogram (ECG) from bipolar chest leads was measured before the CNV paradigm and after the arithmetic task in each task set (Fig. 4). The R-R interval obtained from the ECG data was analyzed by the FFT technique with MaP1060 Ver5.16 (Nihonsanteku Co., Ltd., Osaka, Japan) and the powers of the high-frequency (HF, 0.20–0.35 Hz) and low-frequency (LF, 0.05–0.20 Hz) components were calculated to evaluate autonomic nervous activity. LF/HF and HF/(LF + HF) were considered measures of sympathetic and parasympathetic nerve tones, respectively.

#### Melatonin

Saliva samples obtained with a Salivette saliva collector (Sarstedt AG & Co. KG, Sarstedt, Germany) were centrifuged for 5 min at 1000 rpm, frozen at – 20 °C, and stored until assayed. The melatonin levels of the samples were analyzed in duplicate with a commercially available RIA kit (Direct Saliva Melatonin RIA, RK-DSM2; BÜHLMANN Laboratories AG, Schönenbuch, Switzerland). The mean values of duplicates were used for the analysis.

#### Subjective evaluation

The questionnaires used in Canazei et al. [18] consisted of 38 room atmosphere-related terms, 6 room lighting-related terms, and 1 item of connectedness to nature. Our study used the same questionnaires of room lighting and connectedness to nature and selected 7 of the 38 room atmosphere-related terms that were found to have significant main effects of light and time by Canazei et al. [18].

Each questionnaire is detailed below.

1. Room atmosphere questionnaire

- Seven atmosphere terms: threatening, cozy, active, uninhibited, safe, restless, and stimulating
- Rating categories and numerical coding: 1, “not at all”; 2, “a little”; 3, “rather”; and 4, “very”

2) Room lighting questionnaire

- Six room lighting-related bipolar adjective pairs: pleasant–unpleasant, attractive–unattractive, natural–artificial, glary–without glare, dim–bright, and even–uneven
- Numerical ratings from 0 to 5

3) One item of connectedness to nature

- Rating categories and numerical coding: 1, “not at all”; 2, “a little”; 3, “rather”; and 4, “very”

### Statistical analysis

Types of light sources and time were used as the variables of each measurement, and two-way analyses of variance were conducted with Bonferroni correction using SPSS version 22.0 (SPSS Inc., Chicago, IL, USA). Differences with *p* < 0.05 were considered statistically significant.

## Results

**Fig. 6:**
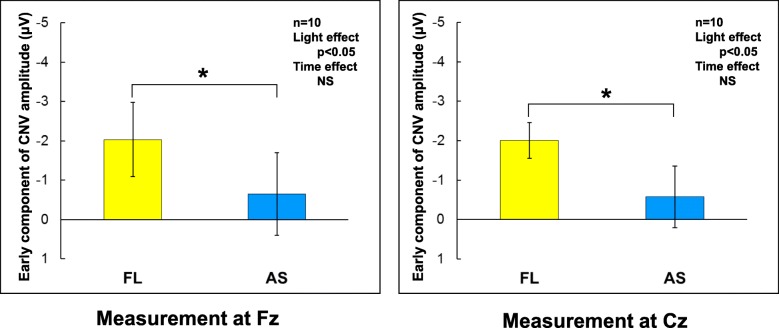
Early component of CNV amplitude obtained from Fz and Cz. A larger negative value indicates higher arousal. AS, artificial skylight; FL, fluorescent lights

Brain arousal levels were evaluated by the *α*-wave ratio and early component of the CNV amplitude. The *α*-wave ratio is the arousal level in response to non-specific multiple stimuli around participants, whereas the early component of the CNV amplitude is the arousal level in response to specific serial events occurring during the CNV paradigm. There was no significant difference in the *α*-wave ratio between the two lighting conditions. However, the early components of CNV amplitudes were significantly higher at both Fz and Cz under the fluorescent lights than under the artificial skylights (Fig. 6; both *p* < 0.05) with no time variation.

Regarding productivity measured by the arithmetic task (number of answers and ratio of correct answers) and the selective response time obtained during the CNV paradigm, no significant differences were observed. Lighting conditions had no effect on calculation and selective response times that required concentration, cognition, and judgment.

**Fig. 7:**
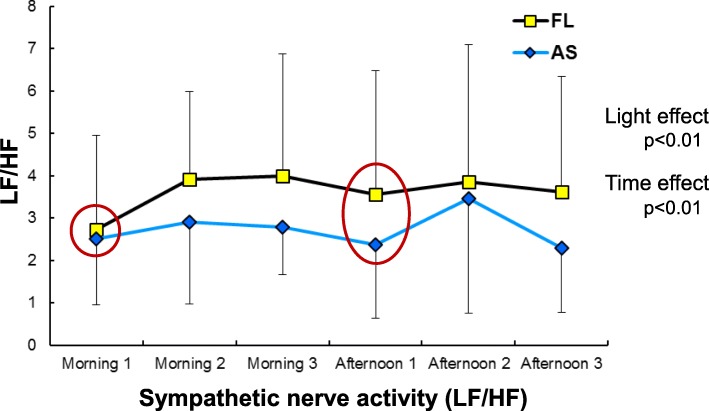
Sympathetic nervous activity (LF/HF) evaluated from heart rate variability (HRV). AS, artificial skylight; FL, fluorescent lights

**Fig. 8:**
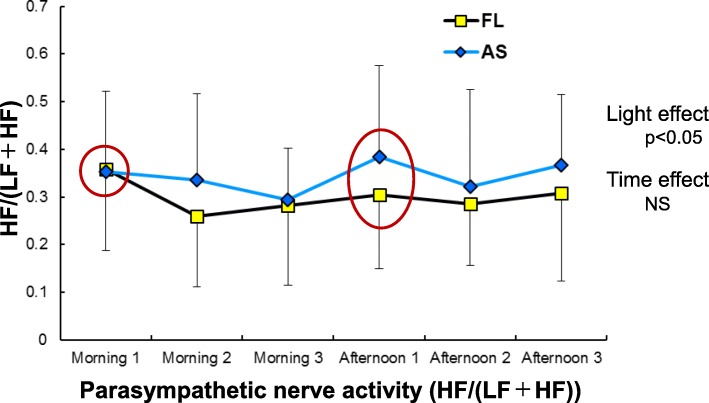
Parasympathetic nervous activity (HF/(LF+HF)) evaluated from heart rate variability (HRV). AS, artificial skylight; FL, fluorescent lights

Sympathetic and parasympathetic nervous activities, obtained from HRV, are shown in Figs. 7 and 8, respectively. Sympathetic nervous activity for overall measurements in the daytime indicated significant effects of light and time (both *p* < 0.01). Although there was no difference before the first task in the morning (morning 1 in Fig. 7), the difference became more evident throughout the day and was still present at afternoon 1 before the first task in the afternoon, showing that the activity was significantly lowered under the artificial skylights compared with the fluorescent lights. In the same manner, a significant effect of light on parasympathetic nervous activity was observed, with enhanced activity under the artificial skylights compared with the fluorescent lights (*p* < 0.05).

**Fig. 9:**
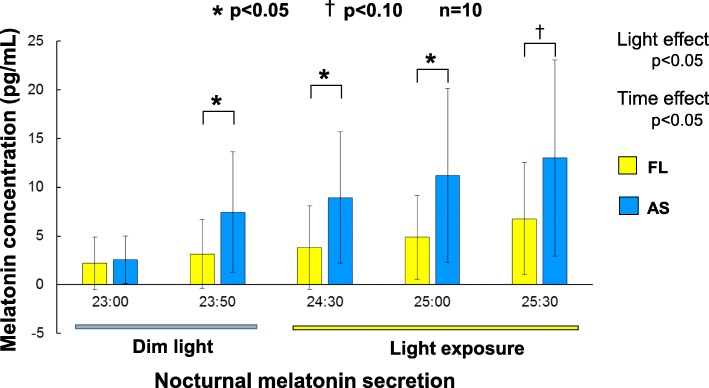
Nocturnal melatonin secretion during dim light and light exposure. AS, artificial skylight; FL, fluorescent lights

Nocturnal melatonin secretion is shown in Fig. 9. Melatonin secretion was measured at 23:00 and 23:50 under dim light conditions (< 30 lx) and at 00:30, 01:00, and 01:30 during fluorescent light exposure (500 lx and 5000 K). There were significant main effects of light and time (both *p* < 0.05). According to multiple comparisons by Bonferroni correction, no difference was found at 23:00. However, a significant difference was evident 50 min later under dim light conditions; this difference was greater in the participants exposed to the artificial skylights (*p* < 0.05). The difference continued even during light exposure.

**Fig. 10:**
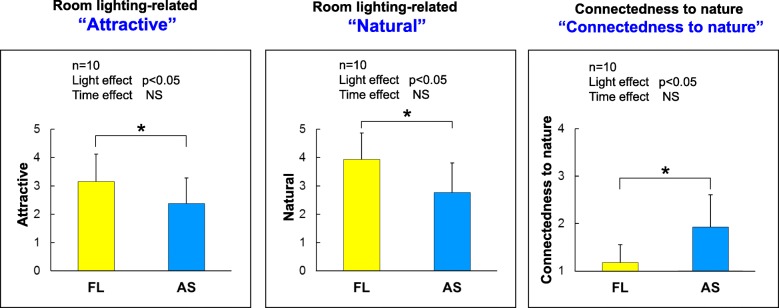
Subjective evaluations. The vertical scales of left and middle graphs show that lower values are more attractive and natural, respectively, and the right shows that higher value indicates more connectedness. AS, artificial skylight; FL, fluorescent lights

Regarding subjective responses, there were significant main effects of light both for room lighting-related effects and connectedness to nature, showing that participants considered artificial skylights more “attractive” and more “natural” (both *p* < 0.01) than fluorescent lights among the 6 items of room lighting-related effects and also felt greater connectedness to nature (*p* < 0.01) (Fig. 10). However, no significant changes with time were found for all three subjective items.

## Discussion

There has been considerable focus on green construction and environmental sustainability in recent years, but relatively little attention has been paid to biological sustainability. Studies of occupant health and well-being in built environments have recently begun to be conducted. It has been demonstrated that light, in addition to air and water quality, has a strong effect on occupant health and well-being in offices, not only from the point of view of subjective comfort, but also in terms of objective aspects or non-visual effects [15, 19, 21, 33].

A newly developed artificial skylight was examined here, although its spectral distribution is not exactly the same as that of natural sunlight. The aims of this study were to examine the non-visual effects of lights in terms of cortical arousal levels, autonomic nervous activity, and nocturnal melatonin secretion; to examine work productivity and subjective responses during diurnal exposure to artificial skylights and conventional fluorescent lights in a simulated office environment; and to evaluate whether artificial skylights should be favored in offices from the viewpoint of adaptability to diurnal light.

### Subjective evaluations

Canazei et al. [18] were the first to perform subjective comparisons of artificial skylights and fluorescent lights in a simulated office environment built in a laboratory. They investigated subjective responses by using questionnaires, which consisted of 38 room atmosphere-related terms, 6 room lighting-related terms, and 1 item of connectedness to nature. Our study used the same items related to room lighting and connectedness to nature, and we selected 7 of the 38 room atmosphere-related items found to exert significant main effects of light and time in Canazei et al. The artificial skylight in their study was much larger size and had a higher light intensity (1215 lx) than our skylights (400 lx). However, significant effects were obtained from two terms—“attractive” and “nature”—of perceived room lighting and connectedness to nature; no significant effects were found among the 7 items of perceived room atmosphere in our study. Thus, these 3 items showing significant effects even at around 400 lx might be the features of skylights that mimic natural blue sky with indefinite depth.

### Non-visual effects

Many studies have focused on the non-visual effects of light [7, 34], with application studies showing its beneficial effects on human health and well-being and how to translate scientific findings into office lighting designs [15, 19, 22, 23]. Non-visual responses include almost all physiological functions affected by photic signals from ipRGCs projected to all major retinorecipient parts of the brain [28, 35]. From the viewpoint of the relationship between CCT and the action factors on circadian rhythm, non-visual responses are dependent on the physical nature of light, such as its intensity and spectral distribution, and not on the type of light source [36]. In this study, the non-visual effects of artificial skylights and conventional fluorescent lights during daytime exposure were evaluated in terms of arousal level, autonomic nervous activity, and nocturnal melatonin secretion. The arousal level of the brain and autonomic nervous tension are thought to be associated with office work productivity. Nocturnal melatonin secretion, which affects sleep and circadian rhythms, might also have an indirect effect on long-term work performance.

Regarding arousal levels, the *α*-wave ratio and the early component of the CNV amplitude were measured. The *α*-wave ratio was obtained from spontaneous EEG immediately before the CNV paradigm was begun and represents the arousal level with non-specific stimulation. The CNV, one of the event-related potentials, has been evaluated as expectancy [32] and motivation [37] while the early component of the CNV amplitude has been considered the attention or arousal level with the specific serial events of warning, imperative stimuli, and button pushing [38]. This type of attention is called the phasic arousal level [39].

### Arousal level or alertness

There have been many studies on alertness involving spontaneous EEG evaluation. These studies have reported that alertness and cortical arousal level were increased by monochromatic blue light and daylight with a higher CCT in the case of polychromatic light [40–44]. However, there was no significant difference in the *α*-wave ratio in this study between the two types of light studied, showing that the arousal level with non-specific stimulation was the same under the two lighting conditions.

On the other hand, the early component of the CNV amplitude or the phasic arousal level measured at both Fz and Cz was significantly lower under the artificial skylights than under the fluorescent lights. It might be speculated that the artificial skylights would increase sleepiness and lower work productivity. However, there was no increase in the *α*-wave ratio reflecting increased sleepiness. In addition, the work performance of the selective response time and arithmetic task did not reveal any differences between the two lighting conditions. The results suggested that individuals under artificial skylights would have the same work performance as those under fluorescent lights with a lower phasic arousal level and same sleepiness. In other words, occupants under the fluorescent lights might have a slightly excessive tension of arousal level when achieving the same work compared with those under the artificial skylights [45].

Deguchi and Sato [46] were the first to report the effect of the CCT of light on the early component of the CNV amplitude. They evaluated phasic arousal levels under CCT conditions of 3000 K, 5000 K, and 7500 K with the same light intensity of 1000 lx. Their results showed that the simple response time during the CNV paradigm was not significantly affected by CCT while the early component of the CNV amplitude was significantly higher with 7500 K of light than with 3000 K. Subsequent studies also demonstrated the same results, namely, that a higher CCT enhanced the arousal level obtained by the CNV paradigm [47, 48]. Because ipRGCs are sensitive to short-wavelength light, a higher CCT with a greater amount of short-wavelength energy is considered to strengthen non-visual effects [26, 28, 30]. However, the phasic arousal level was significantly lower in the participants exposed to the artificial skylights than in those exposed to the fluorescent lights, even though the CCT and light intensity of the lights measured at the occupant’s eye level in this study were higher under the artificial skylights (417 lx with 6200 K) than under the fluorescent lights (330 lx with 4800 K). This finding is different from that of other studies.

The non-visual effect is supposed to be affected not only by direct photic stimulation to ipRGCs, but also integrated signal input to ipRGCs from rods and S, M, and L cones in the retina. Because the integration process is not completely understood, the non-visual effects cannot be entirely explained by the intensity and CCT of light [30, 49]. It is implied that the results obtained with the different spectral distributions of the two light sources in this study could be affected by differences in the integration of signals from the five photic receptors. However, the cause is unknown. Different types of light sources were used in this study, whereas many other studies have used the same light source to specifically examine CCT and/or intensity effects. Therefore, one of the possible reasons for the conflicting result might be an impact on the psychological impression of the artificial skylights. Non-visual effects were originally considered independent of visual effects or psychological responses to a view, and yet, it was very likely that the psychological effects of an artificial skylight with no glare and indefinite depth would reduce tension to a moderate level because the skylight gives occupants comprehensive feelings of a natural atmosphere and connectedness to nature. Thus, although the differences in the light sources themselves were believed to not affect non-visual effects [36], the impact on psychological factors might overlap with non-visual functions.

### Autonomic nervous activity

In terms of autonomic nervous activity, it is generally considered that sympathetic nerve tone becomes dominant over parasympathetic tone immediately before and during mental work. Some studies have examined the effect of the CCT of light on autonomic nervous activity evaluated using HRV [50, 51], blood pressure [52], and body temperature [53, 54]. These studies reported that a higher CCT strengthens sympathetic nerve tone or both sympathetic and parasympathetic tones.

In the case of autonomic nerve tone related to cardiac activity evaluated from HRV in this study, the sympathetic nerve tone (LF/HF) was significantly lower under the artificial skylight conditions than under the fluorescent light conditions when all of the daytime data were pooled (*p* < 0.01; Fig. 7). Similarly, parasympathetic tone (HF/(LF+HF)) was significantly higher with the skylight (*p* < 0.05; Fig. 8). Thus, our results indicate lower autonomic nerve tension with the skylight than with the fluorescent lights, despite the higher CCT of the skylight. This finding also conflicts with those of previous reports. As shown in Figs. 7 and 8, both sympathetic and parasympathetic nerve tones were similar for the two types of lights immediately before the first CNV paradigm at morning 1; the tension in the fluorescent light condition tended to increase at mornings 2 and 3, with the tendency continuing into the afternoon and being even higher at afternoon 1 immediately before the CNV task. These results suggest that consistent work performance with less tension, evaluated not only from the early component of the CNV amplitude but also from HRV, could be maintained with the artificial skylights, despite the higher CCT of skylights. These findings again imply that differences in psychological responses might have affected non-visual effects, as seen in the phasic arousal level and autonomic nervous activity.

### Nocturnal melatonin secretion

Although few studies have investigated the effects of light exposure in daytime on non-visual responses at night, these reports have shown effects on nocturnal melatonin secretion [55], dim light melatonin onset (DLMO) [56], and sleep quality [57, 58]. Therefore, it was expected that the melatonin secretion measured in this study would also provide information on how different light sources in daytime affect nocturnal melatonin secretion, DLMO, and sleep quality. It was anticipated, for example, that the more endogenous the melatonin secretion before sleep, the greater the improvement in sleep quality [59–62]. The increased melatonin secretion before sleep might be caused by advanced DLMO or secretion activation by light exposure in the morning. The previous studies showed that the higher intensity and/or CCT of light in the morning, the smaller the light-induced nocturnal melatonin suppression and the more advanced the circadian rhythm phase (DLMO) [15, 55, 63]. There might be psychological effects that occurred in daytime due to different light sources. However, only fluorescent lights were used at night when melatonin secretion was evaluated. If any differences in melatonin secretion were found, they would be caused specifically by the difference in physical features of light sources used in the daytime. The melatonin secretion was measured at 23:00 and 23:50 (dim light condition < 30 lx) before participant exposure to fluorescent lights, with the results indicating that melatonin secretion at 23:50 was significantly increased under daytime skylight conditions than under fluorescent conditions; no difference in melatonin secretion between the two lighting conditions was observed at 23:00 (Fig. 9). Furthermore, the melatonin secretion obtained at 00:30 and 01:00 during light exposure at 500 lx with 5000 K was found to be significantly higher in those exposed to the artificial skylights than in those exposed to the fluorescent lights, with the tendency maintained until 01:30 (Fig. 9). The melanopic illuminance was 4.99 melanopic lx with skylights, which is about 35% greater than the 3.70 melanopic lx with fluorescent light. In addition, cyanopic, rhodopic, chloropic, and erythropic illuminances were also 22%, 22%, 8%, and 0% higher, respectively, with the skylight. It is unclear how interactions of photoreceptive units (cones [L, M, S], rods, and ipRGCs) activate ipRGCs. However, the following features of ipRGCs are known [30]: (1) the fundamental light response is an irradiance-dependent increase in firing, (2) they are less sensitive than rods or cones (which means greater stimulation is needed), and (3) once the threshold for ipRGC activation has been reached, activation is remarkably persistent over a long duration of constant illumination. Therefore, it is speculated that, in addition to higher CCT with skylight, these integrated photoreceptor inputs might contribute to increased nocturnal melatonin secretion and/or an advanced DLMO shift. Our findings suggest that the use of artificial skylights in office environments might help to maintain a constant circadian rhythm when advanced DLMO actually occurs because the circadian rhythm is generally slightly longer than 24 h and ordinary exposure to light at night additionally delays DLMO. Our results also suggest that skylights have a weak effect on light-induced nocturnal melatonin suppression even with a higher light intensity (500 lx) than that found in ordinary homes. This might also contribute to sleep improvement, as demonstrated by our previous polysomnography study showing that higher melatonin secretion before sleep could lead to a longer period of stage 4 sleep [58].

## Conclusions

Artificial skylights that simulate natural sunlight and blue skies help to maintain appropriate work performance with lower psychological and physiological stress and promote consistent long-term work productivity with comfort and good health from the point of view of increased nocturnal melatonin secretion and/or an advanced shift in circadian rhythms.

## Data Availability

The datasets used and/or analyzed during the current study are available from the corresponding author on reasonable request.
